# Supplementary material for: Semaphorin4A-Plexin D1 Axis Induces Th2 and Th17 While Represses Th1 Skewing in an Autocrine Manner
Source: Int J Mol Sci. 2020 Sep 22;21(18):6965. doi: 10.3390/ijms21186965 (PMC7555002; doi:10.3390/ijms21186965)
Supplement: Supplementary file 1 [file ijms-21-06965-s001.pdf]

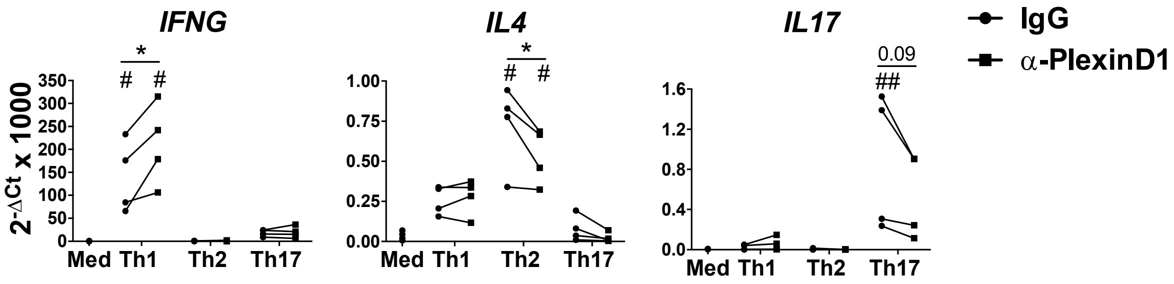

Supplementary Figure S1. Sema4A-PlexinD1 axis inhibits Th1 and promotes Th2 and Th17 differentiation. Cytokines mRNA expression in naïve CD4^{+} T cells differentiated either in medium (Med), Th1, Th2 or Th17 differentiation cocktails in the presence of an anti-PlexinD1 antibody or its respective isotype control for 3 days (n=4). Data is presented as connected dots. * p<0.05. # p<0.05 and ## p<0.01 compared to medium.

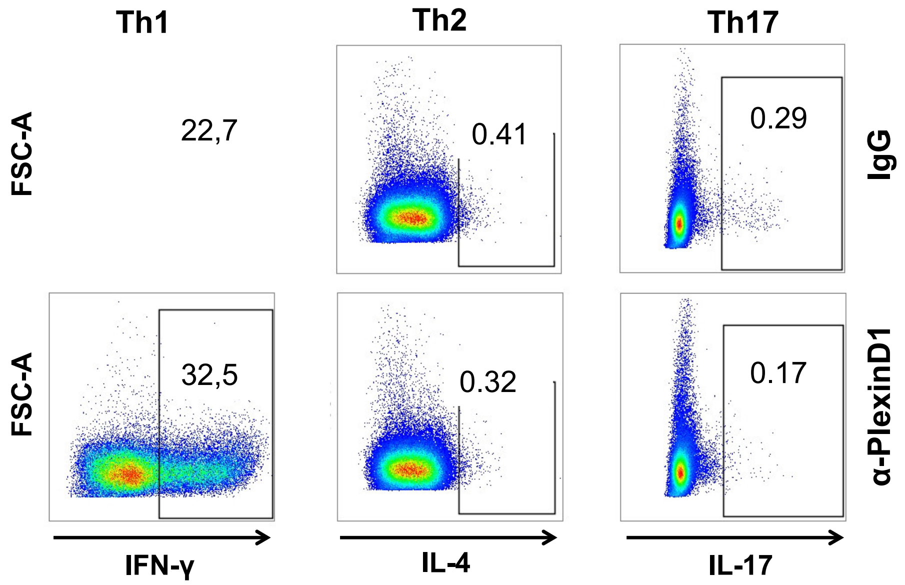

Supplementary Figure S2. Sema4A-PlexinD1 axis inhibits IFN-γ and promotes IL-4 and IL-17 production. Representative plots of intracellular cytokine production in naïve CD4^{+} T cells differentiated with Th1, Th2 or Th17 differentiation cocktails in the presence of an anti-PlexinD1 antibody or its respective isotype control for 7 days.

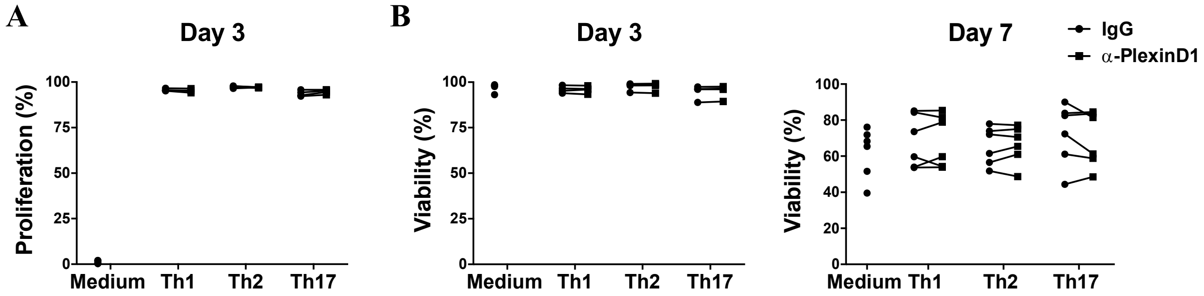

Supplementary Figure S3. Sema4A-PlexinD1 axis blockade does not affect cell proliferation and viability. Cell proliferation (A) and viability (B) in naïve CD4$^+$ T cells differentiated in medium or Th1, Th2 or Th17 differentiation cocktails in the presence of an anti-PlexinD1 antibody or its respective isotype control for 3days (n=4) or 7 days (n=6). Data is presented as connected dots.

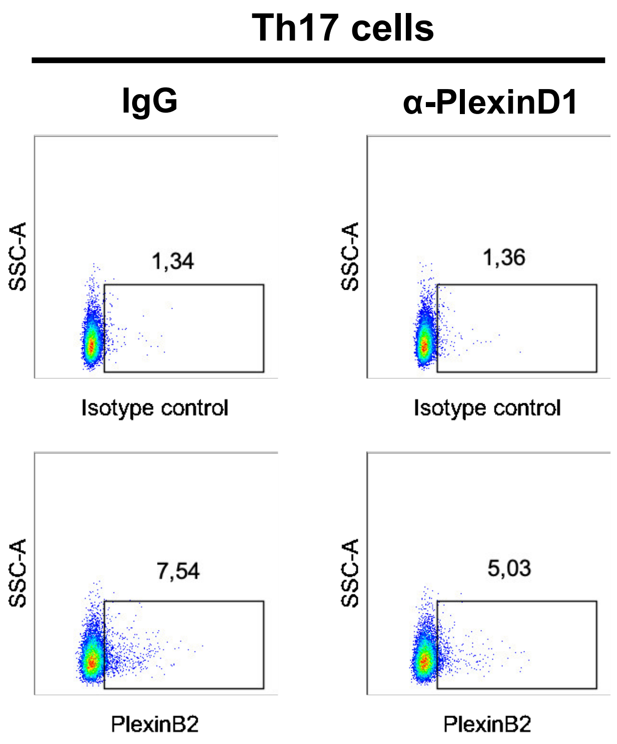

Supplementary Figure S4. Sema4A-PlexinD1 axis inhibition reduces the expression of PlexinB2 in Th17 cells. (A) Representative flow cytometry plot of PlexinB2 expression in Th17-differentiated CD4$^+$ T cells in the presence of an anti-PlexinD1 antibody or its respective isotype control for 3 days.

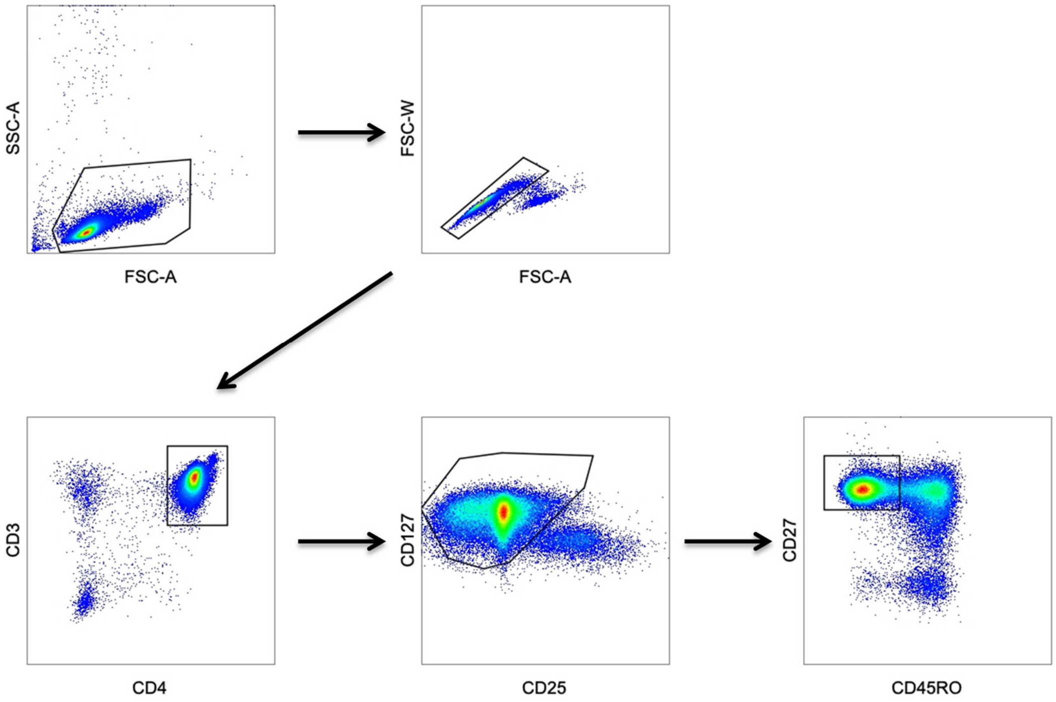

Supplementary Figure S5. Analysis strategy of sorted naïve $CD4^+$ T cells. Flow cytometry gating strategy analysis to sort naïve $CD4^+CD25^-CD27^+CD45RO^-$ T cells.

Supplemental Table S1. List of T helper cell differentiation cocktails.

**Th1 differentiation**	**Company**	**Concentration**
IL-12	Biolegend	5 ng/ml
Anti-IL4	R&D systems	1 µg/ml

**Th2 differentiation**	**Company**	**Concentration**
IL-4	R&D systems	10 ng/ml
Anti-IFNγ	R&D systems	1 µg/ml

**Th17 differentiation**	**Company**	**Concentration**
IL-1β	R&D systems	10 ng/ml
IL-6	Immunotools	10 ng/ml
IL-23	R&D systems	10 ng/ml
TGF-β1	Biolegend	10 ng/ml
Anti-IL4	R&D systems	1 µg/ml
Anti-IFNγ	R&D systems	1 µg/ml

Supplementary Table S2. Characteristics of SSc patients.

	SSc (n=8)
Age (years)	66 (58-72)
Female: n (%)	6 (75)
Disease duration (years)	7 (4.5-8.5)
ANA positive: n (%)	6 (75)
ACA positive: n (%)	6 (75)
Scl70 positive: n (%)	1 (12.5)
mRSS	5 (1-9)
DMARDs: n (%)	1 (12.5)
Biologicals: n (%)	0 (0)

Data is presented as the median (interquartile range) or number (percentage). ANA: antinuclear antibodies; ACA: anticentromere antibodies; Scl70: antitopoisomerase antibodies; mRSS: modified Rodnan Skin score; DMARDs: disease-modifying antirheumatic drugs.

Supplemental Table S3. List of primers.

Gene	Primer forward	Primer reverse
IL17	5’CCGTGGGCTGCACCTGTGTC3’	5’GGGAGTGTGGGCTCCCCAGA3’
IL22	5’GCTGGCTAAGGAGGCTAGCTT3’	5’CATACTGACTCCGTGGAACAGTTT3’
IFNγ	5’ GCAAGGCTATGTGATTACAAGGC3’	5’GTGAAATAAACACACAACCCATGG3’
TNF	5’TCTTCTCGAACCCCGAGTGA3’	5’CCTCTGATGGCACCACCAG3’
IL4	5’AGCAGTTCCACAGGCACAAG3’	5’ACTCTGGTTGGCTTCCTTCAC3’
IL5	5’ACTGCTTTCTACTCATCGAACTCTG3’	5’TTGACTCTCCAGTGTGCCTATTC3’
TBX21	5’TCCAACACGCATATCTTTACTTTCC3’	5’AGCTGAGTAATCTCGGCATTCTG3’
GATA3	5’CAGACCACCACAACCACAC3’	5’TGCCTTCCTTCTTCATAGTCAG3’
RORC	5’AAGACTCATCGCCAAAGCAT3’	5’TCCACATGCTGGCTACACA3’
PLEXIND1	5’GGCCGAGTGAAAGACTTGGA3’	5’GGTGAGACTTCTTGGGCTCC3’
PLEXINB1	5’CTGCAGAAGTTCGTGGATGA3’	5’GAGGCAAGCTGTTGGTCTTC3’
PLEXINB2	5’TCTCAGTCAAGGGCACACTG3’	5’GCGGTAAGCTGTTCGTCTTC3’
NRP1	5’GAAAAATGCGAATGGCTGAT3’	5’AATGGCCCTGAAGACACAAC3’
ILT4	5’TGCTGATCTGAGTCTGCCTG3’	5’CTTGGGGATGGTCCCTGTCT3’
B2M	5’GATGAGTATGCCTGCCGTGT3’	5’TGCGGCATCTTCAAACCTCC3’
